# Fontan Route Remodeling over Time: A Longitudinal Quantitative 3D Case Series

**DOI:** 10.3390/jcdd13010045

**Published:** 2026-01-13

**Authors:** Raquel dos Santos, Amartya Dave, Mohammed Usmaan Siddiqi, Aashi Dharia, Deqa Muse, Junsung Kim, Kameel Khabaz, Nhung Nguyen, Luka Pocivavsek, Narutoshi Hibino

**Affiliations:** 1 Section of Cardiac Surgery, Department of Surgery, The University of Chicago, Chicago, IL 60637, USA; raqueldossantos@uchicago.edu (R.d.S.); ad3qx@umsystem.edu (A.D.); dmuse@uchicago.edu (D.M.); juliuskim1012@uchicago.edu (J.K.); 2 School of Medicine, University of California San Francisco, San Francisco, CA 94143, USA; amartya.dave@ucsf.edu; 3 Molecular, Cellular & Developmental Biology, Pierson College, Yale University, New Haven, CT 06520, USA; usmaan.siddiqi@yale.edu; 4 Department of Radiology, Division of Interventional Radiology, David Geffen School of Medicine, University of California Los Angeles, Los Angeles, CA 90095, USA; kkhabaz@mednet.ucla.edu; 5 Section of Vascular Surgery, Department of Surgery, The University of Chicago, Chicago, IL 60637, USA; nhungng@bsd.uchicago.edu (N.N.); lpocivavsek@bsd.uchicago.edu (L.P.)

**Keywords:** Fontan procedure, congenital heart disease, single-ventricle physiology, remodeling, somatic growth, conduit properties, 3D modeling, shape analysis, growth mapping, cardiovascular biomechanics

## Abstract

Fontan patients experience anatomical remodeling over time, yet the mechanisms driving these changes remain unclear. This study aimed to characterize full-route Fontan remodeling and evaluate whether observed morphological changes arise from somatic growth alone or from the combined influence of conduit properties, surgical design, thoracic anatomy, and mechanical forces. Five Fontan patients (four extracardiac, one lateral tunnel) underwent analysis using two MRI-derived 3D models obtained between 1 and 4 years apart. Directional displacement was assessed using 3D shape overlays, surface geometry was quantified using the Koenderink Shape Index (KSI), and patient-specific growth mapping estimated localized tissue dynamics. Statistical analyses included a one-sample *t*-test for mean anterior displacement, the Grubbs’ test for outlier detection, and the Wilcoxon signed-rank test for KSI comparisons across time points. All patients exhibited anterior displacement of the Fontan route, with a mean shift of 0.29″ ± 0.33″ and one significant outlier (lateral tunnel, 0.87″). Four of five patients showed increased convexity over time. Growth mapping revealed minimal, heterogeneous native-tissue expansion, with localized growth up to 0.2 mm/year. Individual remodeling trajectories varied and did not consistently align with localized anterior growth, indicating that Fontan route remodeling is highly individualized and cannot be explained by somatic growth alone. This retrospective longitudinal case series study highlights the value of quantitative 3D geometric tools for assessing subtle Fontan route remodeling and supports the feasibility of growth-aware, patient-specific modeling frameworks in single-ventricle physiology.

## 1. Introduction

The Fontan procedure, first introduced in 1971 as a palliative surgical intervention for pediatric patients with single-ventricle congenital heart defects, revolutionized management of complex cardiac anatomies by redirecting systemic venous blood directly to the pulmonary circulation, bypassing the functionally single ventricle [1]. Modern iterations typically employ an extracardiac (EC) conduit—constructed from non-growing polytetrafluoroethylene (PTFE) material—to connect the inferior vena cava (IVC) and the pulmonary artery (PA), bypassing the right atrium and establishing the Fontan route [1]. Another iteration of the procedure utilizes a lateral tunnel design, which incorporates native atrial tissue. In this work, the Fontan route refers to the conduit-PA complex.

The procedure has markedly improved survival, with 90% of patients reaching age 30 and 80% reaching age 40, aided by advancements in surgical technique and refined patient selection [2,3]. The IVC-PA connection helps reduce atrial distension, arrhythmia, and thrombosis [3].

Despite these improvements, substantial morbidity and long-term complications persist among those with unfavorable hemodynamics [3]. Surgically implanted conduits may develop stenosis or dilation over time [3]. As the Fontan population ages, risks increase for Fontan failure and premature death, including worsening systemic ventricular function and elevated pulmonary vascular resistance [1]. Long-term complications remain prevalent, though rates vary by patient age, Fontan type, and duration of follow-up. In one large cohort study, 20% of patients experienced arrhythmias, 7% thromboembolism, and 21% required reintervention [2]. Furthermore, adolescents and young adults with Fontan circulation report lower quality of life compared to healthy controls, specifically in physical and psychosocial functioning [4].

A key driver of these complications is conduit-growth mismatch—the synthetic EC graft is non-growing, whereas native tissues (e.g., PA and atrial tissue in LT) continue to enlarge with somatic growth. In this study, growth refers to native-tissue surface expansion. Any EC conduit change reflects deformation within a growing thorax, not material growth. Computational models show that this biomechanical mismatch can elevate wall shear stress by up to 300% at PA-conduit junctions, thereby promoting adverse remodeling [5]. Clinical data also suggest growth asymmetry: in a retrospective analysis of 574 Fontan patients, pre-pubertal height z-scores averaged near normal (−0.2 for boys, −0.1 for girls) but declined significantly post-puberty (−0.7 for boys, −0.5 for girls) to produce adult height deficits of 5 cm in boys and 4.5 cm in girls [6]. Such growth disparities create unique challenges for rigid conduits, as native PA segments continue to expand while synthetic grafts remain fixed. Accordingly, 78% of conduits implanted before age 5 develop flow-limiting stenoses by adolescence, correlating with a 2.4-fold increase in Fontan failure risk [2]. The Australian and New Zealand Fontan Registry (ANZFR) further reinforces the importance of this problem, reporting that only 41% of patients remain free of serious cardiovascular events by age 40 [2].

Hemodynamic consequences of conduit mismatch are multifaceted. Computational Fluid Dynamics (CFD) simulations reveal that undersized conduits create velocity mismatches at the IVC–conduit junction, with mean velocities rising from 11 cm/s in the IVC to 23 cm/s in the conduit, corresponding to a 2.1-fold increase in energy loss, particularly in flattened geometries [7]. Four-dimensional flow MRI analyses similarly demonstrate that undersized EC conduits generate accelerated flow with elevated wall shear stress and increased viscous energy loss [7].

Predictive power remains limited. In vitro validation studies with 3D-printed Fontan models show that CFD predictions of hepatic flow distribution vary significantly with mesh resolution (*p* = 0.0002) [5]. Similarly, a self-powered Venturi conduit lowered venae cavae pressures by 0.7 mmHg in patient-specific models yet redistributed only 30% of flow to the left PA compared with 70% in idealized models [8]. Together, these findings highlight how existing simulation frameworks often fail to capture growth-mediated remodeling and patient-specific variability.

Recent work suggests that the mismatch between expected and observed remodeling reflects a combination of biomechanical and molecular influences on somatic growth and vascular adaptation. For instance, dysregulation of insulin-like growth factor (IGF-1) has been linked to higher brain natriuretic peptide (BNP) levels and reduced systemic flow in Fontan patients [9]. Additionally, the use of rigid, non-native conduit materials has been associated with adverse pulmonary vascular remodeling, characterized by medial thinning and eccentric intimal fibrosis [10].

To address these gaps, we analyzed longitudinal MRI-derived 3D models from five Fontan patients using shape overlays, Koenderink Shape Index (KSI), and surface-based growth mapping to evaluate remodeling in relation to somatic growth and the mechanical environment. The purpose of this study was to assess the feasibility of using longitudinal MRI-derived 3D shape analysis and quantitative, patient-specific growth mapping to characterize full-route, individualized Fontan remodeling over time. Rather than determining definitive mechanisms, this preliminary work aimed to evaluate whether these tools could detect subtle geometric changes, visualize localized growth patterns, and provide supplemental insight beyond conventional diameter or cross-sectional measurements.

## 2. Materials and Methods

This study is a retrospective longitudinal case series designed to evaluate the feasibility of quantitative 3D geometric tools for detecting subtle Fontan route remodeling, rather than to establish definitive mechanistic conclusions. We analyzed five patients who underwent the Fontan procedure, each with two deidentified Magnetic Resonance Imaging (MRI) scans obtained between 1 and 4 years apart (Table 1). The scan intervals were one year for Patients 1 and 3, two years for Patients 2 and 4, and four years for Patient 5 (Table 1). These longitudinal datasets enabled intra-patient assessment of morphological and geometric changes in the Fontan route and inter-patient comparisons of growth rates. Patients 1–4 underwent the extracardiac (EC) Fontan procedure using non-compliant PTFE conduits, whereas Patient 5 received a lateral tunnel (LT) Fontan incorporating native atrial tissue (Table 1).

Institutional Review Board approval was not required because all imaging data were deidentified and analyzed retrospectively.

### 2.1. Image Segmentation for 3D Model Generation

Patient-specific geometries were extracted from axial MRI images using Simpleware ScanIP (S-2021.06-SP1; Synopsys, Mountain View, CA, USA). Segmentation of the Fontan conduit and the proximal branch pulmonary artery (PA) was performed using a semi-automated process combining intensity thresholding and manual refinement to delineate anatomical boundaries. Anatomical contours were approximated with solid disks to generate a closed 3D surface shell. Surface smoothing with Gaussian and mean-field filters was applied, followed by meshing to generate high-resolution 3D models of each patient’s Fontan route [11]. Models were exported in Abaqus Input File (.inp) and MAT-file (.mat) formats for downstream analysis.

### 2.2. Shape Overlay for Morphological Comparisons

The 3D shape overlay method aligns two patient-specific models from different time points and measures the maximal shift of the Fontan route. It provides an intuitive visualization and a simple quantitative estimate of directional displacement, though it only captures a single displacement measure and cannot describe regional variations in shape.

Each patient’s models were imported into Abaqus CAE (2021; Dassault Systèmes, Waltham, MA, USA) and aligned to a standardized orientation for morphological displacement analysis. Models were reoriented such that the anterior surface faced left, posterior surface right, the PA superior, and the Fontan conduit inferior. Each patient’s geometry from each time point was overlaid for visualization, with the first time point (green) positioned in front and the second time point (red) positioned behind.

Morphological displacement was quantified by aligning the inferior edges of both models to a common horizontal plane and placing a yellow arrow across the maximal anterior shift of the Fontan route. Arrow length (in inches) represented the displacement between the first and second time points for each patient.

To complement the qualitative observations, a one-sample *t*-test was used to assess whether the mean displacement differed significantly from zero. Grubbs’ test was applied to identify statistical outliers.

### 2.3. Computational Shape Analysis with Koenderink Shape Index (KSI)

The Koenderink Shape Index (KSI) is a scale-invariant metric that characterizes local surface shape using a bounded scale from fully concave (−1), flat (0), ideal cylinder (0.5), to fully convex (+1) [12]. This provides a continuous descriptor of surface geometry that can be compared across time and between patients, allowing detection of subtle changes in surface shape that diameter or CSA alone may miss. However, it requires high-quality segmentation and is sensitive to local surface noise.

KSI was computed for each 3D mesh using a custom MATLAB (2023b, MathWorks, Natick, MA, USA) script developed by Khabaz et al. (2024) [13]. At each mesh vertex, the two principal curvatures (κ_1_, κ_2_) were calculated using the Rusinkiewicz algorithm to approximate the shape operator as a weighted average of adjacent face curvatures. KSI was defined as:

$$KSI=\frac{2}{\pi }arctan\frac{\kappa_{1}+\kappa_{2}}{\kappa_{1}-\kappa_{2}}(\kappa_{1}\geq \kappa_{2})$$

For each model, the mean and variance of KSI values were calculated to characterize surface morphology. Percent change in mean KSI between time points quantified geometric remodeling over time, with positive values indicating increased convexity.

To test whether KSI changes were consistent across patients, paired values from the two time points were compared using the Wilcoxon signed-rank test. A two-sided *p* < 0.05 was considered statistically significant.

### 2.4. Patient-Specific Growth Mapping

To further characterize morphological remodeling, a patient-specific growth mapping algorithm was applied based on the morphoelastic model by Khabaz et al. (2024) [13]. This framework compares two 3D surface models of the same patient at different time points, using finite element (FE) analysis to estimate how much each small region has expanded, contracted, or remained stable [13]. This method can reveal heterogeneous remodeling patterns along the entire Fontan route but is computationally intensive and infers surface area change without distinguishing true somatic growth from non-growing graft deformation.

Preprocessing was performed in MATLAB and FE simulations were performed in Abaqus CAE. For each patient, the 3D geometries of the first and second time point meshes were globally transformed and spatially aligned using rigid and non-rigid registration [13]. Aligned geometries were subdivided into surface partitions, and localized area changes were calculated between corresponding regions on the two geometries [13]. Per-partition growth rates were calculated as the proportional area change between corresponding regions with a linear time approximation [13].

This yielded growth rates in millimeters per time, where “time” corresponded to the duration between time points [13]. Positive values represented localized expansion and negative values represented localized shrinkage. Together, the rates defined a patient-specific growth field describing how much each region expanded, contracted, or remained stable.

To minimize discretization noise, the growth field was smoothed using K-nearest neighbors to ensure continuous surface variation [13]. The resulting growth field was input into a morphoelastic FE model in Abaqus [13]. Importantly, growth was assumed to be isotropic with a constant growth rate and neo-Hookean material properties to focus on geometry-driven remodeling rather than material-specific effects [13].

Finally, growth rate heatmaps (mm/year) were generated to visualize geometric change across the route. Local surface area change is estimated irrespective of tissue type. Because the EC graft is non-growing, changes along the graft surface were interpreted as deformation rather than material growth, whereas positive values along native tissues (e.g., PA and atrial tissue in LT) represent native-tissue expansion. Color gradients were scaled by patient to highlight regions of maximal growth (red), no change (green), and shrinkage (blue). Standard alignment was used for intra-patient comparisons of localized growth over time.

## 3. Results

### 3.1. Shape Overlay for Morphological Comparisons

**Figure 1:**
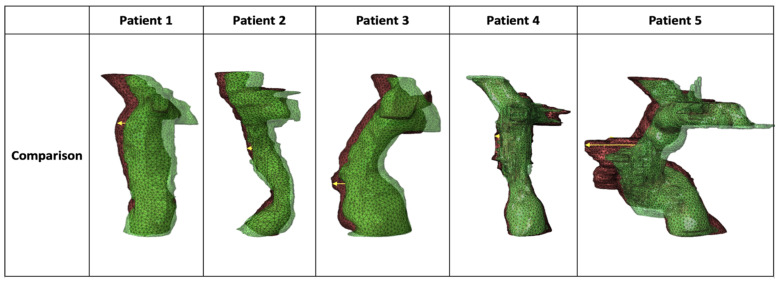
3D shape overlay of the Fontan route comparing the first time point (green) and second time point (red). A yellow arrow marks the direction and magnitude of anterior displacement at the region of greatest change.

Shape overlay analysis enabled qualitative intra-patient comparisons of Fontan route morphology over time. All five patients exhibited a consistent anterior displacement of the Fontan route, evident in the overlay images where the second time point (red) was shifted leftward relative to the first time point (green), with yellow arrows measuring the region of greatest change (Figure 1). Patient 5, who underwent the LT procedure, showed the most pronounced anterior displacement (Figure 1). Because Patient 5 includes native atrial tissue as part of the LT pathway, its region of maximal anterior displacement may reflect remodeling of native tissue rather than PTFE conduit deformation and is therefore interpreted as a structurally distinct case.

Quantitative analysis of anterior displacement yielded values of 0.15″, 0.10″, 0.23″, 0.09″, and 0.87″ for Patients 1–5, respectively. The mean displacement was 0.29″ ± 0.33″. A one-sample *t*-test indicated that the mean did not differ significantly from zero (t = 1.95, *p* = 0.123). However, Grubbs’ test identified Patient 5’s displacement of 0.87″ as a statistically significant outlier (G = 1.76 > G_crit_ = 1.72, *p* < 0.05).

### 3.2. Computational Shape Analysis with Koenderink Shape Index (KSI)

**Figure 2:**
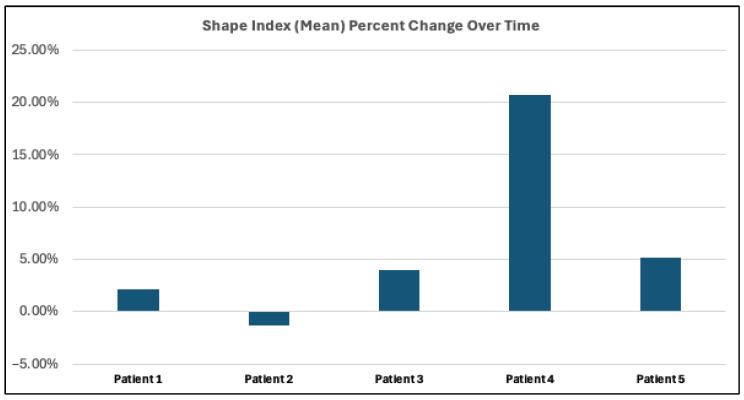
Mean KSI percent changes by patient, with positive values indicating increased convexity over time and negative values indicating decreased convexity.

KSI analysis provided a quantitative assessment of surface geometry between time points. Four out of five patients exhibited a positive percent change in mean KSI values, indicating a general shift toward increased convexity over time (Figure 2). Percent changes ranged from 2.14% to 20.72%, reflecting inter-patient variability in geometric remodeling (Figure 2). One patient showed a slight negative change of −1.31%, indicating minor localized flattening (Figure 2).

**Figure 3:**
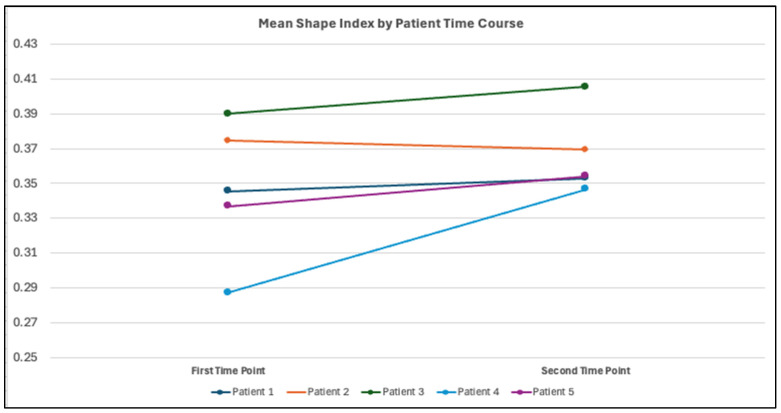
Mean KSI values for each patient at both time points.

Across all time points, mean KSI values ranged from 0.2871 to 0.4056, corresponding to moderately convex morphologies between flat (0) and ideal cylinder (0.5) geometries (Figure 3). Variance in KSI ranged from 0.1133 to 0.1767, indicating relatively low within-model shape variability.

Wilcoxon signed-rank testing (two-sided) showed a trend toward increasing convexity (median ΔKSI = +0.0157, *p* = 0.0625). Although not statistically significant, the result supports the observed remodeling trajectory.

### 3.3. Patient-Specific Growth Mapping

**Figure 4:**
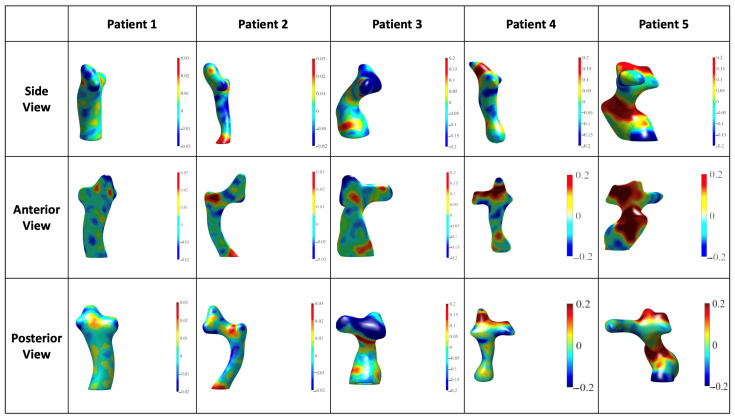
Growth mapping visualization via heatmaps (mm/year) with three views of each model. Color gradients were scaled by patient to maximize resolution and highlight areas of greatest change. Dark blue indicates shrinkage, green indicates no change, and red indicates maximal growth.

Growth mapping revealed heterogeneity in both magnitude and spatial distribution of localized growth across patients. While all patients exhibited anterior morphological shifts over time, these displacements were not consistently associated with elevated anterior native-tissue growth. Patient 5 (LT) showed the highest localized anterior growth. In contrast, anterior growth was negligible for Patients 1 and 2 and minimal for Patients 3 and 4, all of whom underwent EC Fontan procedures (Figure 4). This suggests that the common anterior remodeling trajectory cannot be explained by localized somatic growth alone and is also influenced by conduit properties, surgical design, thoracic anatomy, and the mechanical environment.

Patients 3, 4, and 5 exhibited the most pronounced full-route growth, with maximum expansion rates reaching up to 0.2 mm/year (Figure 4). In contrast, Patients 1 and 2 exhibited modest full-route growth, with peak values of 0.03 mm/year (Figure 4). Shrinkage was present in all patients, as indicated by dark blue heatmap regions (Figure 4). The spatial distribution of shrinkage varied, further reinforcing the individualized nature of Fontan route remodeling.

## 4. Discussion

This study provides a preliminary, feasibility-focused characterization of directional displacement, surface geometry, and surface-based growth of the Fontan route using longitudinal MRI-derived 3D models. Analysis of five patients over 1 to 4 years revealed that Fontan route remodeling is highly individualized and multifactorial. All patients demonstrated anterior displacement and four of five showed increased convexity over time, although the spatial distribution and magnitude of these changes varied. Importantly, growth mapping revealed no consistent pattern of localized anterior native-tissue growth, suggesting that somatic growth alone does not fully explain the observed remodeling trajectories.

Prior studies of Fontan remodeling have largely relied on conventional diameter or cross-sectional area (CSA) metrics. Lee et al. (2007) and Patel et al. (2021) examined proximal or distal conduit changes using singular or averaged CSA values, which do not capture full-route geometry or directional displacement [14,15]. Restrepo et al. (2015) reported variable diameter growth in LT pathways and no size changes in EC pathways but did not evaluate directional remodeling or underlying mechanisms [16]. More recently, Lee et al. (2024) attributed conduit area loss to structural degradation and compression, particularly in oversized conduits, but did not assess full-route evolution [17]. By integrating 3D shape overlays, computational shape analysis, and patient-specific growth mapping, our multimodal approach provides a more comprehensive framework for evaluating Fontan remodeling beyond traditional measurements.

Mean anterior displacement across the cohort was 0.29″ ± 0.33″, which did not differ significantly from zero (t = 1.95, *p* = 0.123). However, the Grubbs’ test identified the LT patient (Patient 5) as a statistically significant outlier with a displacement of 0.87″. This suggests that the magnitude of displacement observed in this patient is unlikely to represent random inter-patient variability and instead reflects the behavior of the LT Fontan, which incorporates native atrial tissue and may permit greater dilation and remodeling than the rigid, non-compliant PTFE conduits used in the EC procedures of the other four patients.

Koenderink Shape Index (KSI) analysis demonstrated a cohort-wide trend toward increasing convexity, with four of five patients showing positive percent changes over time. This pattern suggests progressive geometric reshaping of the Fontan route surface that may influence local hemodynamics and differentiate patient-specific remodeling trajectories. Because KSI is scale-invariant, these changes reflect true alterations in surface shape rather than size alone, further emphasizing the importance of geometric characterization beyond diameter-based metrics.

Interpretation of growth mapping requires distinction between biologic tissue growth and mechanical deformation. The EC grafts used in four patients are made of non-growing PTFE material. Accordingly, positive growth values along the graft surface reflect deformation within a growing thorax rather than material growth, whereas positive values along the pulmonary arteries and the LT atrial tissue represent native-tissue growth.

Growth mapping revealed heterogeneity in both magnitude and spatial distribution of localized expansion across patients. Although all patients exhibited anterior displacement, these shifts were not consistently associated with elevated localized anterior native-tissue growth. Patient 5 (LT) showed the highest anterior growth, while anterior growth was negligible or minimal in the four EC patients. Full-route expansion varied widely, from modest (0.03 mm/year) to more pronounced increases (0.2 mm/year). Shrinkage was observed in all patients. This disconnect between displacement and localized growth reinforces that anterior remodeling cannot be attributed to somatic growth alone, but instead reflects the combined influence of conduit properties, surgical design, thoracic anatomy, and the mechanical environment.

Given the inclusion of only one LT patient, no comparative conclusions regarding Fontan type can be drawn. Instead, the LT case illustrates the sensitivity of the geometric framework to distinct tissue compositions and biomechanical environments.

These findings highlight a key limitation of the EC Fontan: synthetic conduits cannot grow in tandem with native vasculature, particularly the pulmonary arteries. As somatic growth progresses through childhood and adolescence, this mismatch may drive geometric distortion and progressive remodeling of the Fontan route that compromises performance. This emphasizes how conduit material properties and surgical design shape long-term remodeling trajectories.

A likely explanation for the consistent anterior displacement observed across patients is anatomical constraint imposed by the thoracic cavity. The Fontan conduit is typically positioned along the posterior thoracic wall, where limited space restricts posterior expansion. As patients grow, this constraint may drive preferential anterior remodeling, where more thoracic space is available. Additionally, the mechanical mismatch between rigid grafts and dynamic somatic tissues may promote conduit skewing, torsion, and asymmetric stress distributions. Prior studies have reported conduit compression by adjacent thoracic structures, supporting this mechanical interpretation [14,17].

Although localized native-tissue surface expansion—specifically the pulmonary arteries and the LT atrial tissue in Patient 5—was minimal in several patients, somatic growth may still indirectly contribute to Fontan remodeling through changes in thoracic geometry, altered loading conditions, and spatial constraints that influence conduit deformation and displacement.

Taken together, our findings suggest that Fontan remodeling is not a uniform consequence of somatic growth, but rather reflects a complex interplay among conduit properties, surgical design, thoracic anatomy, and the mechanical environment. These results highlight that somatic growth alone cannot predict long-term Fontan route evolution and underscore the need for patient-specific planning strategies beyond simple conduit sizing.

The inclusion of adult patients allowed for observation of remodeling after the completion of rapid somatic growth, where observed changes are more likely to reflect mechanical deformation or structural remodeling rather than biologic growth. This provided a complementary context for evaluating the sensitivity of the proposed geometric framework.

This study has several limitations. The imaging intervals (between 1 and 4 years) may not capture the full extent of long-term remodeling, particularly through adolescence and early adulthood. The small sample size (*n* = 5) constrains generalizability. The growth mapping algorithm was developed for adult aortic geometries and may not fully account for the complex morphology of pediatric pulmonary vasculature. In addition, although growth heatmaps provided spatial visualization of remodeling trajectories, the analysis was primarily qualitative. The method infers surface area change without distinguishing material growth from deformation changes of non-growing grafts. No statistical comparisons of localized growth distributions were performed across patients, limiting quantification of inter-patient heterogeneity.

Nonetheless, this study provides a key clinical insight: remodeling trajectories and growth rates vary considerably across patients. As shown here, short-term geometric changes of the Fontan route are generally small, making visual or subjective assessment challenging and potentially inconsistent. Quantitative geometric measurement provides objective and reproducible indicators of subtle morphological variation. By detecting trends in shape evolution earlier and more accurately—such as localized narrowing, asymmetric growth, or progressive pathway shift—clinicians may identify unfavorable remodeling patterns that could precede functional deterioration.

Such quantitative information can support more informed patient follow-up planning. When adverse geometric trends are identified, follow-up intervals could be shortened to allow earlier detection of changes associated with Fontan circulation failure. In this way, quantitative geometric assessment may complement conventional qualitative interpretation and enhance clinical decision-making for long-term surveillance.

Future planning tools may benefit from incorporation of predictive modeling that accounts for both somatic growth and the evolving mechanical environment, such as the growth framework described by Khabaz et al. (2024) [13]. By simulating individualized remodeling trajectories from baseline scans, clinicians may be able to virtually test surgical strategies, optimize imaging and intervention timing, and better anticipate long-term complications.

## 5. Conclusions

This study represents a preliminary evaluation of Fontan route remodeling in five patients using longitudinal MRI-derived 3D shape overlays, computational shape analysis, and patient-specific growth mapping. Unlike prior work limited to conduit diameters or cross-sectional areas, our approach captured full-route morphology and directional displacement over time. These findings demonstrate the feasibility of quantitative geometric analysis for detecting subtle Fontan route changes and provide a foundation for future growth-aware, patient-specific modeling frameworks. Further studies with larger, developmentally stratified cohorts will be necessary to confirm these observations and define their clinical relevance.

## Figures and Tables

**Table 1:** Five Fontan patients with two MRI scans taken between 1 and 4 years apart. Diagnoses include HLHS (Hypoplastic Left Heart Syndrome), DORV (Double Outlet Right Ventricle), Pulmonary Atresia, and VSD (Ventricular Septal Defect). Abbreviations include IVC (Inferior Vena Cava) and AV (Atrioventricular).

Patient	Age at Fontan (Years)	Age at 1st MRI (Years)	Age at 2nd MRI (Years)	Diagnosis	Fontan Type
1	2	10	11	HLHS	EC
2	2	23	25	DORV, Pulmonary atresia, VSD, Interrupted IVC	EC
3	2	23	24	Unbalanced common AV canal, Interrupted aortic arch	EC
4	2	15	17	HLHS	EC
5	2	20	24	HLHS	LT

## Data Availability

The original contributions presented in this study are included in the article material. Further inquiries can be directed to the corresponding author.
